# Phenotypic assay for cytotoxicity assessment of *Balamuthia mandrillaris* against human neurospheroids

**DOI:** 10.3389/fmicb.2023.1190530

**Published:** 2023-09-05

**Authors:** Narisara Whangviboonkij, Worakamol Pengsart, Zhenzhong Chen, Seokgyu Han, Sungsu Park, Kasem Kulkeaw

**Affiliations:** ^1^Siriraj Integrative Center for Neglected Parasitic Diseases, Department of Parasitology, Faculty of Medicine Siriraj Hospital, Mahidol University, Bangkok, Thailand; ^2^School of Mechanical Engineering, Sungkyunkwan University, Suwon, Republic of Korea; ^3^Department of Biomedical Engineering, Sungkyunkwan University, Suwon, Republic of Korea; ^4^Institute of Quantum Biophysics, Sungkyunkwan University, Suwon, Republic of Korea

**Keywords:** granulomatous amoebic encephalitis, *Balamuthia mandrillaris*, neurospheroid, cytotoxicity, drug discovery, neglected disease, tropical disease

## Abstract

**Introduction:**

The phenotypic screening of drugs against *Balamuthia mandrillaris*, a neuropathogenic amoeba, involves two simultaneous phases: an initial step to test amoebicidal activity followed by an assay for cytotoxicity to host cells. The emergence of three-dimensional (3D) cell cultures has provided a more physiologically relevant model than traditional 2D cell culture for studying the pathogenicity of *B. mandrillaris*. However, the measurement of ATP, a critical indicator of cell viability, is complicated by the overgrowth of *B. mandrillaris* in coculture with host cells during drug screening, making it challenging to differentiate between amoebicidal activity and drug toxicity to human cells.

**Methods:**

To address this limitation, we introduce a novel assay that utilizes three-dimensional hanging spheroid plates (3DHSPs) to evaluate both activities simultaneously on a single platform.

**Results and discussion:**

Our study showed that the incubation of neurospheroids with clinically isolated *B. mandrillaris* trophozoites resulted in a loss of neurospheroid integrity, while the ATP levels in the neurospheroids decreased over time, indicating decreased host cell viability. Conversely, ATP levels in isolated trophozoites increased, indicating active parasite metabolism. Our findings suggest that the 3DHSP-based assay can serve as an endpoint for the phenotypic screening of drugs against *B. mandrillaris*, providing a more efficient and accurate approach for evaluating both parasite cytotoxicity and viability.

## 1. Introduction

Some protozoan species naturally reside in the environment, while some are amphizoic and capable of adapting to survive in the human body (Haston and Cope, 2023). *Balamuthia mandrillaris* is an environment-dwelling amoeba that causes lethal brain damage, termed granulomatous amoebic encephalitis (GAE). Metabolically active trophozoites of *B. mandrillaris* can be isolated from soil and freshwater (Gompf and Garcia, 2019). Thus, two routes of transmission are proposed: direct exposure to soil and water via skin ulcers or inhalation via the olfactory epithelium in the nose (Kiderlen and Laube, 2004; Piper et al., 2018; Gompf and Garcia, 2019) or the respiratory tract (Schuster and Visvesvara, 2004a). According to the report in 2019, more than 200 cases of GAE have been estimated (Gompf and Garcia, 2019). Although Balamuthia-GAE is extremely rare, the disease is highly fatal (Schuster and Visvesvara, 2004b). More than a 95% mortality rate is documented worldwide. Regardless of immune competency, everyone is at risk of being infected by *B. mandrillaris* (Wu et al., 2020; Xu et al., 2022). Hence, environment-derived *B. mandrillaris* infections have challenged our efforts to prevent this highly lethal disease.

The poor prognosis of GAE is multifactorial. Differential diagnosis of GAE from other inflamed brains is difficult. GAE has symptoms similar to those of viral or bacterial encephalitis, which are more common. Moreover, a definitive diagnosis is based on observation of the trophozoites in the histological section of the inflamed brain under a microscope (Peng et al., 2022; Liu et al., 2023). The sensitivity of the microscopic diagnosis depends on the site of the brain biopsy, the number of trophozoites, and the skill of the examiner. Other laboratory procedures may support definitive diagnosis, including time-consuming *in vitro* culture and/or high-sensitivity detection of trophozoite DNA (Wu et al., 2020; Yang et al., 2020). However, the lack of a sensitive diagnosis makes GAE more dangerous (Schuster and Visvesvara, 2004b). A definitive diagnosis is often made after death by autopsy of brain tissue. Importantly, there is no specific drug targeting *B. mandrillaris*. Current treatments for GAE rely primarily on trial combinations of antimicrobial and antifungal drugs, resulting in variations in clinical outcomes (Doyle et al., 2011; Cuoco et al., 2022). Thus, the development of more effective drugs remains a topic of intensive research (Phan et al., 2021; Ferrins et al., 2023).

Three-dimensional (3D) cell cultures have emerged as useful models for the study of pathogenicity owing to their similarity to cell and tissue physiology compared to conventional 2D cell culture. Several cancer cell lines are capable of forming spherical cell aggregates, known as spheroids (Kapalczynska et al., 2018). Additional physiological relevance of spheroids has been recognized, including cell functions (Lagies et al., 2020) and drug response (Mittler et al., 2017). Previously, our group developed a chemiluminescence assay for assessing the cytotoxicity of *B. mandrillaris* against the human neurospheroid, a 3D cell clump (Pengsart and Kulkeaw, 2022). However, the measurement of ATP, a critical indicator of cell viability, is complicated by the overgrowth of *B. mandrillaris* in coculture with host cells during drug screening, making it challenging to differentiate between amoebicidal activity and drug toxicity to human cells. We previously reported the use of a three-dimensional hanging spheroid plate (3DHSP) to facilitate the formation of spheroids and the separation of unbound and dead cells during cytotoxicity assays using chimeric antigen receptor (CAR) T cells and demonstrated the direct measurement of cytotoxic effects of CAR T cells on spheroids using optical imaging without the need for live and dead fluorescent staining of the cells (Chen et al., 2022). This study adapts the 3D neurospheroid plate by which the growing trophozoites were separated before measuring the ATP level. Thus, it is applicable for screening amoebicidal activity and cytotoxicity in the same setting. This dual phenotypic assay allows the identification of drugs that ameliorate the severity of disease and drugs that are safe in a single step.

## 2. Materials and methods

### 2.1. Culture of a clinical isolate of *Balamuthia mandrillaris* trophozoites

*Balamuthia mandrillaris* trophozoites were isolated from biopsied brain tissue of a human subject who gave consent to participate (COA no. Si806/2020). The experiments involving *B. mandrillaris* trophozoites were approved by the Siriraj Institutional Review Board (COA. no. 146/2022). The detailed protocol was explained in Pengsart et al. (2022). Briefly, small pieces of the biopsied brain were digested with pepsin. After passing through a sterile gauze bandage, the small cell clumps were mixed with DMEM supplemented with 10% heat-inactivated fetal bovine serum (FBS, HyClone, Utah) and subjected to coculture with a monolayer of human lung carcinoma A549 cells (as feeders). The culture medium was renewed every 2–3 days. The floating cells were transferred to a new lot of feeder cells every week. Any cells with a morphology distinct from that of the A549 cells were observed for slow movement of the projecting cytoplasm. Moreover, a small area of the feeder cell-free appearance could indicate the presence of trophozoites. For regular maintenance, *B. mandrillaris* trophozoites were cultured with A549 cells. Prior to studying neurospheroid damage, trophozoites were plated onto a monolayer of human SH-SY5Y cells for 2–3 passages. When the monolayer of SH-SY5Y cells was removed from more than 80% of the surface area, the trophozoites were transferred to a new monolayer of 80–90% confluent SH-SY5Y cells. For human cell-free culture, the trophozoites were maintained in the feeder-free culture (Law et al., 2023). BM-3 medium was used and consisted of peptone (16.4 mM), yeast extract (4 mg/mL), yeast RNA (1 mg/mL), liver digest (10 mg/mL), glucose (5.5 mM), hemin (3 µM), taurine (0.4 mM), 1xMEM vitamin mixture, 1xlipid mixture, and 10% newborn calf serum.

### 2.2. Culture of human lung carcinoma A549 and neuroblastoma SH-SY5Y cells

Human neuroblastoma SH-SY5Y cells were obtained from the American Type Culture Collection (ATCC^®^ No. CRL-2266TM), while human lung carcinoma A549 cells were kindly provided by Prof. Wanpen Chaicumpa. Both cell types were maintained following the ATCC’s instructions. For the SH-SY5Y cells, a 1:1 ratio of ATCC-formulated Eagle’s minimum essential medium (EMEM) (ATCC, Utah) and F12 medium (Gibco, Gaithersburg, MD) was prepared. The complete medium of the human neuroblastoma SH-SY5Y cells is EMEM-F12 medium supplemented with 10% heat-inactivated FBS (HyClone, Utah), herein called complete EMEM-F12 medium. For the A549 cells, 10% FBS-supplemented DMEM was used. Cells were incubated at 37°C in a humidified atmosphere containing 5% CO_2_ and subcultured every 2–3 days or when the cell density reached 60–80% confluence. Cells were detached from the polystyrene well using 0.25% trypsin in 0.5 mM EDTA solution (STEMCELL Technologies, Vancouver, Canada). Viable and nonviable cells were identified using Trypan blue and counted using a hemocytometer under a light microscope. Human neuroblastoma SH-SY5Y cells were subcultured at a 1:10 ratio per well of a 6-well plate.

### 2.3. Formation of human neurospheroids in 3DHSP

**Figure 1:**
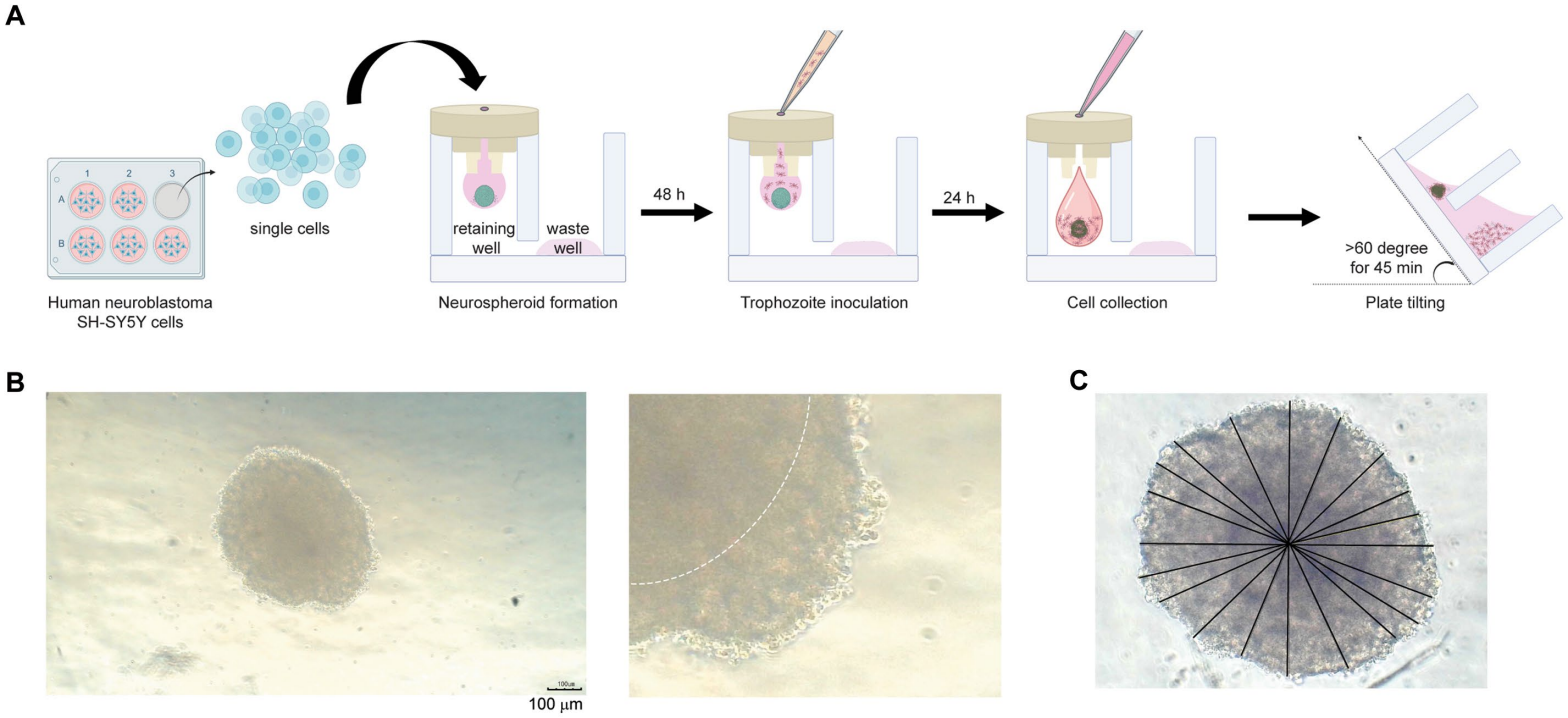
Neurospheroid formation in a three-dimensional hanging spheroid plate (3DHSP). **(A)** Schematic diagram of neurospheroid formation in the 3DHSP. Single cells were hung in a drop in the retaining well, while the waste well was filled with the culture medium. After a 48-h incubation, *B. mandrillaris* trophozoites were incubated with neurospheroids. After 24 h of trophozoite incubation, cells in the hanging drop were collected by adding culture medium. The 3DHSP was tilted at an angle of 60°C for 45 min to separate the trophozoites from the neurospheroids. **(B)** Representative images of human neurospheroids in after 48 h of hanging drop culture. A higher magnification is shown on the right-hand side (scale bar = 100 μm). The dark hypoxic area is marked by the white line. **(C)** Measurement of spheroid diameter. ImageJ was used to measure the diameter. Due to incomplete spherical shape, ten lines are drawn across the neurospheroid, and the lengths of the drawn lines were averaged.

The 3DHSP is a 24-well plate consisting of two layers. The upper layer forms hanging drops, and the lower layer consists of a spheroid retaining well and a waste well connected by channels with a height of 40 μm (Figure 1A). The upper layer was printed using a 3D printer (Cubicon, Seongnam, Korea) with acrylonitrile butadiene styrene (ABS) filaments. The lower layer was made of polydimethylsiloxane (PDMS) (Dow Corning Co., Midland, MI, United States) (Chen et al., 2022). Human neuroblastoma SH-SY5Y cells were collected from the 2D culture well using cell dissociation buffer (Life Technology, New York). Viable cells were prepared at 1.7 × 10^4^ cells per 25 μL of complete EMEM-F12. Then, the cell suspension was filled into a small channel on the upper layer of the 3DHSP. The complete EMEM-F12 was placed into the waste well to prevent evaporation of the hanging drop. The plate was placed in a reservoir containing sterile distilled water and covered with a lid to prevent evaporation of the culture medium in the hanging drop.

### 2.4. Culture of *Balamuthia mandrillaris* with human neurospheroids

After 2 days of hanging drop incubation, 1,000 trophozoites in 10 μL were filled into the same small channel, allowing the coculture of trophozoites with human cells in a hanging drop. For analysis of human cell viability, 100 μL of complete EMEM-F12 medium was added to the hanging drop at 24 h post-coculture to push the cell mixture into the retaining well. The 3DHSP plate was tilted for 45 or 90 min to allow the single cells to pass through a 40 μm-diameter hole. The neurospheroids in the retaining well and the separated cells in the waste well were subjected to analysis of size, morphology, and viability. To measure the diameter of the nonspherical neurospheroid, lines were drawn from the perimeter of the spheroid, passed through the center, and ended at the opposite perimeter. Lengths of the lines were measured using ImageJ software and calculated for a diameter mean.

### 2.5. Fluorescence labeling of human neurospheroids

Human neuroblastoma SH-SY5Y cells were cultured in 2D, and single cells were prepared, followed by incubation with 2.5 μM CellTracker™ Green CMFDA (5-chloromethylfluorescein diacetate, Invitrogen, OR) and 1:1600 diluted DiD (Invitrogen, OR) to label proteins and lipids, respectively. The labeled cells were subjected to neurospheroid formation as mentioned above. After 48 h of hanging drop culture, *B. mandrillaris* trophozoites were added to the hanging drop and incubated for 24 h. The drops were pushed into the lower plate by adding 100 μL of culture medium. The lower plate was tilted at an angle of more than 60° for 45 min at room temperature to separate the cell debris and trophozoites into the waste well. For visualization, neurospheroids and cell debris were transferred into ultralow attachment (SPL Life Sciences, South Korea) and flat-bottom plates (Thermo Fisher Scientific, United States), respectively, for confocal imaging (Nikon Eclipse Ti, Nikon, Japan).

### 2.6. Efficiency of trophozoite removal from human neurospheroids

To examine the separation of the trophozoites from the neurospheroid after 3DHSP tilting, the presence of trophozoites adhering to the neurospheroid in the retaining well and those in the waste well was measured using quantitative PCR. Genomic DNA was isolated using a QIAamp DNA Mini Kit (QIAGEN, Hilden, Germany) following the manufacturer’s instructions. The primer set used for the amplification of 16S rRNA gene of the *B. mandrillaris* included a forward primer (5’-TAACCTGCTAAATAGTCATGCCAAT-3′) and a reverse primer (5’-CAAACTTCCCTCGGCTAATCA-3′) (Wu et al., 2020). The thermal cycles for each transcript were as follows: initial denaturation at 95°C for 1 min, followed by 40 cycles of DNA denaturation at 95°C for 15 s, primer annealing at 60°C for 30 s, and DNA strand extension at 60°C for 5 s. Given the same volume of DNA extracts, the level of *B. mandrillaris* DNA was directly compared among samples using the cycle threshold (Ct). Delta cycle threshold (∆Ct) was calculated following the formula: ∆Ct = [Ct of the trophozoite DNA in the waste well - Ct of the trophozoite DNA in the retaining well] (Rao et al., 2013). The DNA obtained from an untilted plate was set as a positive control because all trophozoites remained. Then, the 2^-∆Ct^ was calculated and displayed as a relative expression compared to the positive control (the untilted plate).

### 2.7. Cell viability

Intracellular ATP was used as a readout indicating the metabolically active stage of viable cells. The CellTiter-Glo^®^ 3D Cell Viability Assay (Promega, United States) was deployed for direct detection of the intracellular level of ATP. ATPs can change luciferin to a luminescence-emitting oxyluciferin. Briefly, the neurospheroids and the separated trophozoites were transferred to a low attachment round-bottom well of the 96-well plate (SPL Life Sciences, South Korea) and a Nunclon Delta Surface 96-well plate (Thermo Fisher Scientific, MA), respectively, followed by adding the CellTiter-Glo^®^ solution. The luminescence signal was measured by using a BioTek Synergy H1 Hybrid Multi-Mode plate reader. After removing the background luminescence of the culture medium, the data were displayed as relative light units (RLUs). Changes in the RLUs of each sample were related to the RLUs of control neurospheroids that were free from *B. mandrillaris* trophozoites.

### 2.8. Statistical analysis

Differences between two independent samples were calculated using the Mann–Whitney test. Student’s *t* test was used to examine the difference in the means of relative DNA and ATP levels and the RLUs of ATP levels. Multiple comparisons of the spheroid diameter of more than two groups were assessed by using one-way ANOVA with Bonferroni correction. A statistically significant difference was dependent on a *p* value: less than 0.05 indicated that a given value of each sample was different, while more than 0.05 implied a probability that a given value was the same among samples.

## 3. Results

### 3.1. Formation of human neurospheroids in the 3DHSP

First, the number of cells was optimized to form a spheroid in a hanging drop. Various numbers of human SH-SY5Y cells were hung as a drop in the upper part of the 3DHSP (Supplementary Figure S1A). To capture images of spheroid-forming cells, culture medium needs to be added to push the cell clump into a well of the lower part, or a stereomicroscope needs to be used to capture the cells in the hanging drop. Thus, early-forming spheroids could not be imaged due to fragmentation. Finally, we determined that the optimal number of human SH-SY5Y cells was 6.8 × 10^5^ cells/mL (Supplementary Figure S1A). At 48 h post cell hanging, the neurospheroid was opaque with central dark zones, and clearly seen edges were observed (dotted line in the right panel, Figure 1B). Given an incomplete spherical shape, several straight lines were drawn from side to side through the center (Figure 1C). The average diameter of the 48-h neurospheroids was 438.4 ± 18 μm (*n* = 6) (Figure 1B). Thus, the human neuroblastoma SH-SY5Y cells could proliferate and form spherical shapes in a hanging drop of the 3DHSP. Notably, sterile distilled water or culture medium was added underneath the hanging drop in the lower part of the plate. Due to proximity to the solution below, some SH-SY5Y cells migrated to adhere to the surface of the lower plate (Supplementary Figure S1B). Thus, the original protocol was modified by placing the 3DHSP in a humidified chamber without the liquid fill in the lower part. Instead, the culture medium was filled in the waste well, preventing the evaporation of liquid from the hanging drop.

### 3.2. Separation of cell debris from the human neurospheroid using 3DHSP

**Figure 2:**
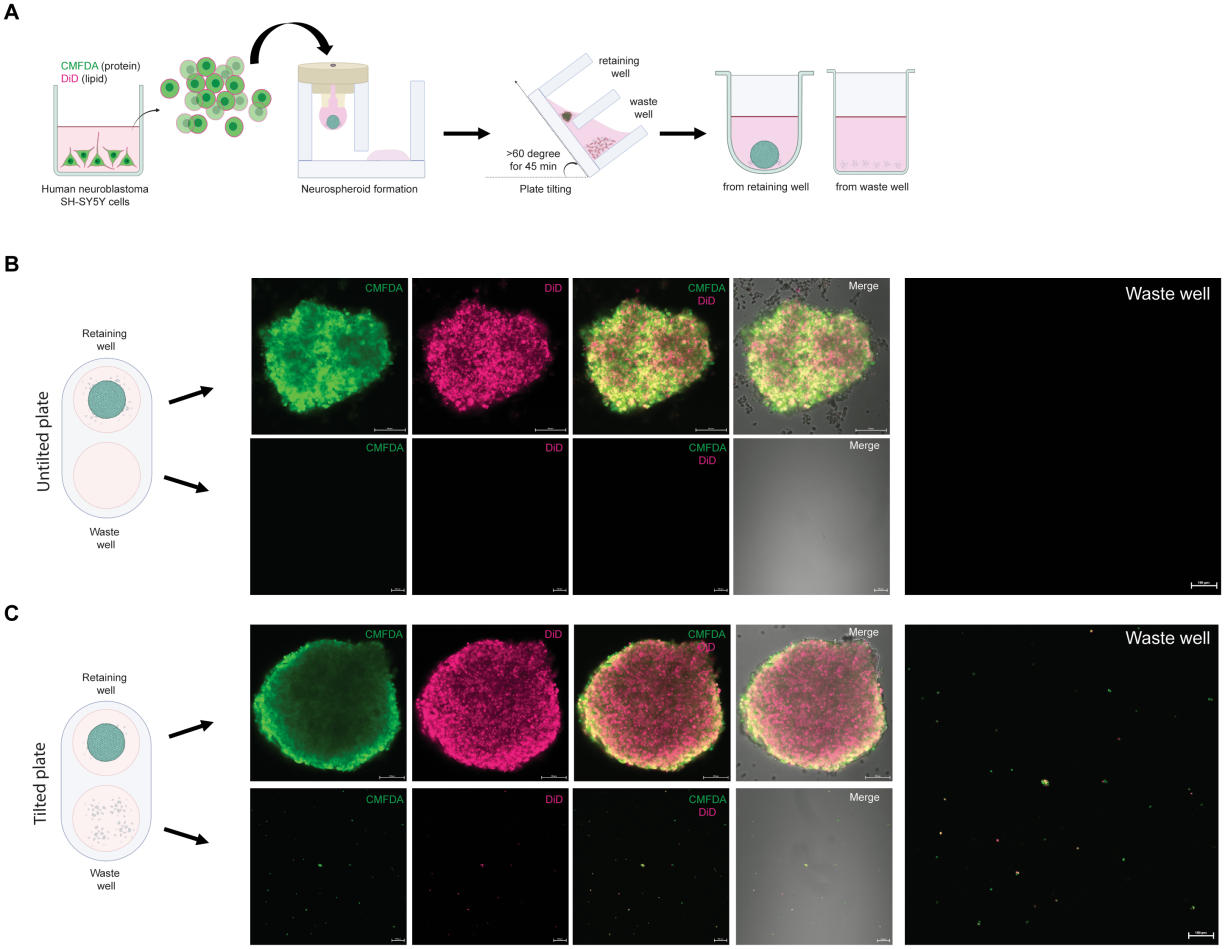
Removal of single SH-SY5Y cells from the neurospheroid. **(A)** Illustration of the method for forming fluorescent neurospheroids. Human neuroblastoma SH-SY5Y cells were incubated with protein-binding CMFDA and lipid-binding DiD in the 2D cell culture well. After cell labeling, single SH-SY5Y cells were subjected to neurospheroid formation in the 3DHSP. After plate tilting, the neurospheroids and the cells in the waste well were transferred to a round or flat bottom-containing plate, respectively. The fluorescence-labeled neurospheroids and cells were imaged under a confocal microscope. **(B)** Confocal microscopic images of SH-SY5Y neurospheroids in the retaining well (upper panel, scale bar = 100 μm) and waste well (lower panel, scale bar = 100 μm) of the untilted plate. Images of CMFDA-and DiD-labeled neurospheroid are merged with the differential interference contrast (Merge). A higher magnification of the CMFDA-and DiD-visualizing waste well is shown on the right-hand side (scale bar = 100 μm). **(C)** Confocal microscopic images of SH-SY5Y neurospheroids in the retaining well (upper panel, scale bar = 100 μm) and the waste well (lower panel, scale bar = 100 μm) after tilting for 90 min. Images of CMFDA-and DiD-labeled neurospheroid are merged with the differential interference contrast (Merge). A higher magnification of the CMFDA-and DiD-visualizing waste well is shown on the right-hand side (scale bar = 100 μm).

An advantage of the 3DHSP is the ability to remove cell debris, as demonstrated previously (Chen et al., 2022). To assess the efficiency of trophozoite separation from neurospheroids, the cells in the retaining and waste wells after plate tilting were visualized. Human neuroblastoma SH-SY5Y cells were labeled with two fluorophores: CMFDA for protein and DiD for lipid (Figure 2A). Following cell labeling, the fluorescence intensity remained detectable for up to 48 h (Figures 2B,C). CMFDA and DiD had different patterns. CMFDA-bound protein was dispersed, while DiD-bound lipids appeared as globules of various sizes. Without plate tilting, the neurospheroids were retained in the chamber. No cell debris was observed in the waste chamber (lower panels, Figure 2B). In contrast, the 60 degree-tilted 3DHSP allowed cell debris to be moved into the waste well (lower panels, Figure 2C). The cell debris observed in the waste well still showed CMFDA and DiD fluorescence. Without fluorescent labeling, it was difficult to observe cell debris using the phase contrast view of the inverted microscope. The representative image shows 47 fluorescent particles per waste well following the 60-degree-tilted 3DHSP while there was no fluorescent particle detected in the waste well without plate tilting.

### 3.3. Loss of neurospheroid integrity in coculture with *Balamuthia mandrillaris*

**Figure 3:**
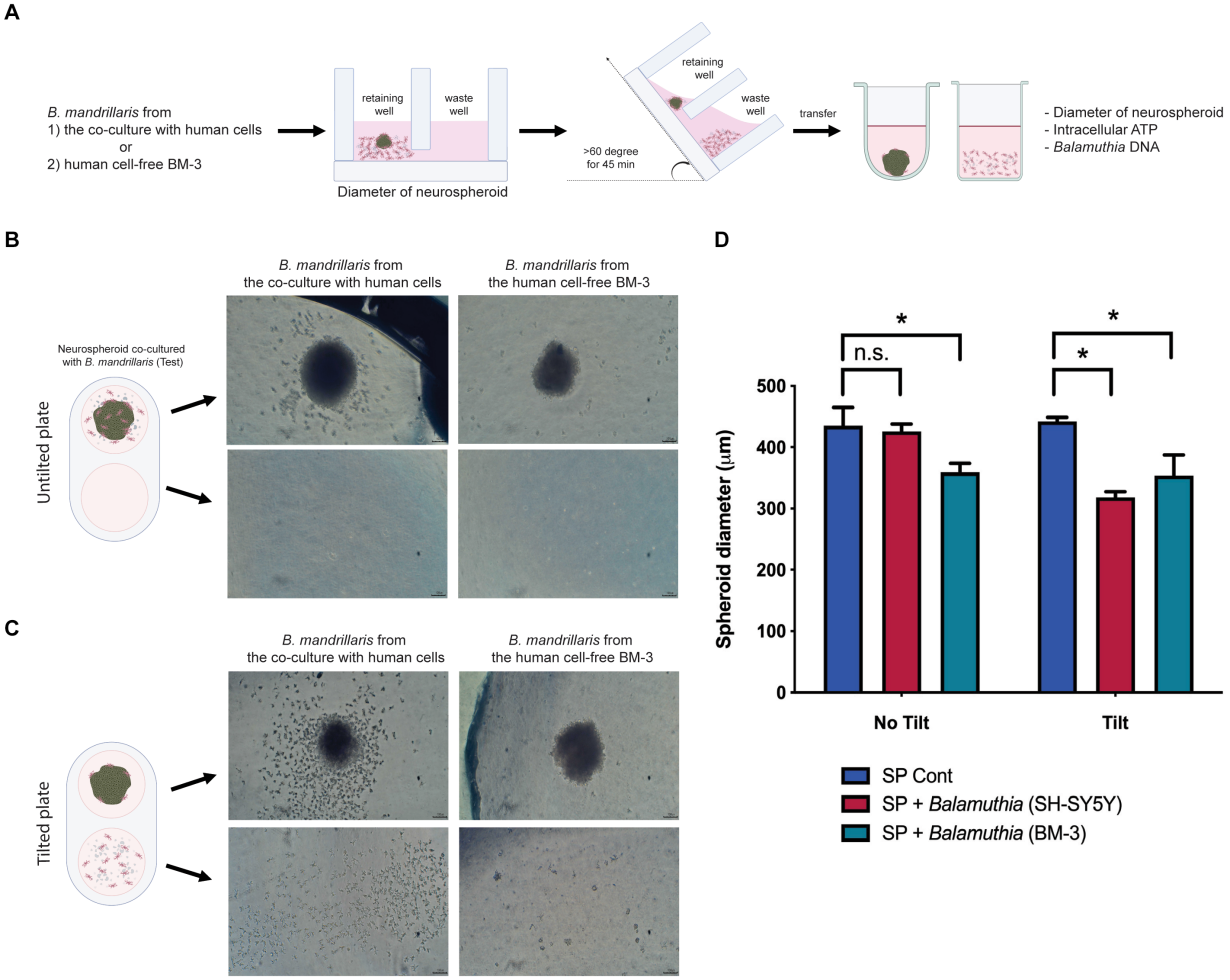
The effects of *B. mandrillaris* trophozoites on the integrity of neurospheroids. **(A)** Schematic diagram illustrating the assessment of the cytotoxicity of trophozoites against neurospheroids. The trophozoites were obtained from two sources: the coculture with human SH-SY5Y cells and the human cell-free BM-3 culture. Following 3DHSP tilting, the neurospheroids and cells in the waste well were transferred into a new round bottom and flat bottom plate for measuring the intracellular ATP and neurospheroid size. **(B)** Illustration of neurospheroids cultured with and without *B. mandrillaris* trophozoites in the hanging drops of the untilted plate. The spherical shape of the neurospheroid in the drop observed under a stereomicroscope. Scale bar, 100 μm. **(C)** Illustration of the neurospheroid and *B. mandrillaris* trophozoites in the retaining well and the waste well after plate tilting. **(D)** Sizes of the neurospheroids in the retaining wells were calculated from the untilted and tilted plates. Due to asymmetry, several lines of diameter were drawn and subjected to the calculation of spheroid diameter using ImageJ (left panel). The bar graph is the diameter of the neurospheroid (right panel). The *B. mandrillaris* trophozoites used in the coculture with neurospheroid (SP) were from the culture with human neuroblastoma cells (SH-SY5Y) or the feeder-free culture (BM-3).

To apply an optical measurement for examining neurospheroid integrity, the diameter of neurospheroids was measured after the settling of spheroids in the retaining well (Figure 3A). Since the trophozoites could be cultured with or without a monolayer of human neuroblastoma SH-SY5Y cells, we examined both types of trophozoites regarding their effects on neurospheroid integrity (Supplementary Figure S2A). The trophozoites were collected after the monolayer of human neuroblastoma SH-SY5Y cells disappeared. After 48 h of coculture with the neurospheroids, some trophozoites adhered to the neurospheroids, while some did not (Figure 3B). Different morphologies of the trophozoites were observed to relate to the source of the trophozoites. Some trophozoites obtained from the monolayered SH-SY5Y cells scattered as single cells, while some adhered to the neurospheroid (Supplementary Figure S2B). In contrast, most of the trophozoites obtained from the feeder-free culture (BM-3 medium) formed clumps proximal to the neurospheroid (Supplementary Figure S2C). Without plate tilting, the sizes of the neurospheroids cocultured with the feeder-derived trophozoites were not different from those of the noninfected neurospheroids (red bar in the untilted plate in Figure 3D). In contrast, the BM3-derived trophozoites significantly reduced the size of the neurospheroid (teal bars, Figure 3D). Following plate tilting, the cell debris and trophozoites were separated into the waste well (Figure 3C). The size of the neurospheroids cocultured with feeder-derived trophozoites was significantly decreased (red bar in tilted plate, Figure 3D). When cocultured with trophozoites from BM-3 medium, the neurospheroids were significantly smaller than the control neurospheroids in both tilted and untilted plates (teal bars, Figure 3D). In summary, a size-based assay allows the assessment of neurospheroid damage in coculture with trophozoites. The culture method used to prepare trophozoites affects this size-based assay.

### 3.4. Decrease in intracellular ATP in coculture with *Balamuthia mandrillaris*

**Figure 4:**
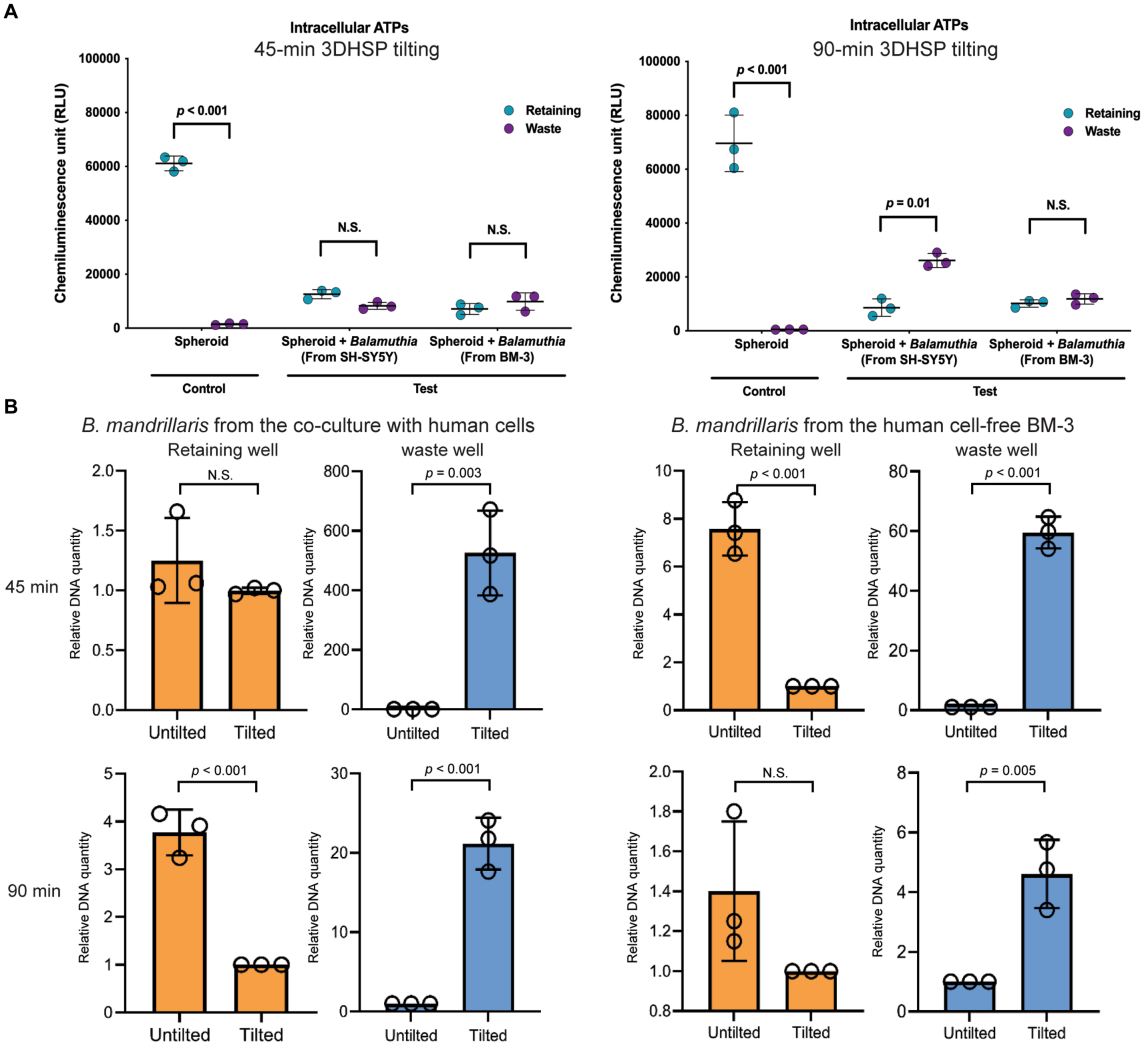
The effects of *B. mandrillaris* trophozoites on the survival of neurospheroids. **(A)** The level of intracellular ATP produced by the neurospheroid in the retaining wells and the *B. mandrillaris* trophozoites in the waste wells. The control group consisted of human neurospheroids without trophozoites, while the test groups consisted of human neurospheroids cocultured with trophozoites. The 3DHSPs were tilted for 45 and 90 min (left and right panels, respectively). Each colored dot represents three biological replicates. **(B)** PCR data show the relative DNA quantity of *B. mandrillaris* DNA remaining in the retaining and waste wells before and after 3DHSP tilting for 45 and 90 min (upper and lower panel, respectively). Trophozoites were obtained from a culture with human neuroblastoma SH-SY5Y cells (left panel) and a feeder-free BM-3 medium (right panel). Circles represent triplicate wells of the quantitative PCR.

After plate tilting, the neurospheroids in the retaining well and the cells in the waste well were transferred into new round bottom and flat bottom plates, respectively (Figure 3A). To examine the survivability of human neurospheroids in coculture with *B. mandrillaris* trophozoites, intracellular ATPs of cells were measured in the retaining well, while those of the trophozoites were measured in the waste well after tilting the 3DHSP for 45 and 90 min (left and right panel, Figure 4A). In the absence of trophozoites, the 3DHSP-derived neurospheroids remained intact in the retaining wells, as indicated by the ATP level (teal dots in control groups in left and right panel, Figure 4A). Nevertheless, the 3DHSP enabled the removal of cell debris, as shown by detectable ATP in the waste well, albeit at the lowest level (dark purple dots, Figure 4A). After coculture with trophozoites and 45-min plate tilting, the levels of intracellular ATPs in neurospheroids were significantly lower by 6-fold than those in noninfected neurospheroids (teal dots in the test groups, Figure 4A). Due to the presence of the trophozoites in the waste wells, the ATP levels increased after plate tilting (dark purple dots, Figure 4A), suggesting a separation of active trophozoites from the neurospheroids. There was no difference in ATP levels between the remaining and waste wells when the 3DHSP was tilted for 45 min; however, a longer tilting time allowed more trophozoites to be separated from the neurospheroids when cocultured with the SH-SY5Y-derived *B. mandrillaris* (right panel, Figure 4A). However, the intracellular ATP level does not discriminate trophozoites, small cell clumps or single cells. To confirm the separation of the trophozoites, PCR was performed to detect the DNA of trophozoites in a relative quantitative manner. As shown in Figure 4B, the DNA of the trophozoites in the retaining well was not changed after 45 min of tilting (orange bars in upper panel, Figure 4B). In contrast, longer 3DHSP tilting (90 min) allowed greater removal of the trophozoites from the neurospheroids (orange bars in lower panel, *p* < 0.001, Figure 4B). Plate tilting significantly increased the number of trophozoites in the waste wells as well (blue bars in the upper and lower panel, Figure 4B). Moreover, the use of trophozoites obtained from feeder-free culture (right panel, Figure 4B) allowed higher efficiency in the separation of the trophozoites from the neurospheroid after 45-min 3DHSP tilting (orange bars in the upper right panel, Figure 4B). In all experiments, the number of trophozoites in the waste well significantly increased following plate tilting (blue bars in all panel, Figure 4B). Regardless of the source of the trophozoites, the 90-min 3DHSP tilting removed the trophozoites from the neurospheroid (blue bars in all panel, Figure 4B), confirming the microscopic imaging (Figure 3C) and ATP detection in the waste wells.

### 3.5. Cytophagy of the *Balamuthia mandrillaris* trophozoites

**Figure 5:**
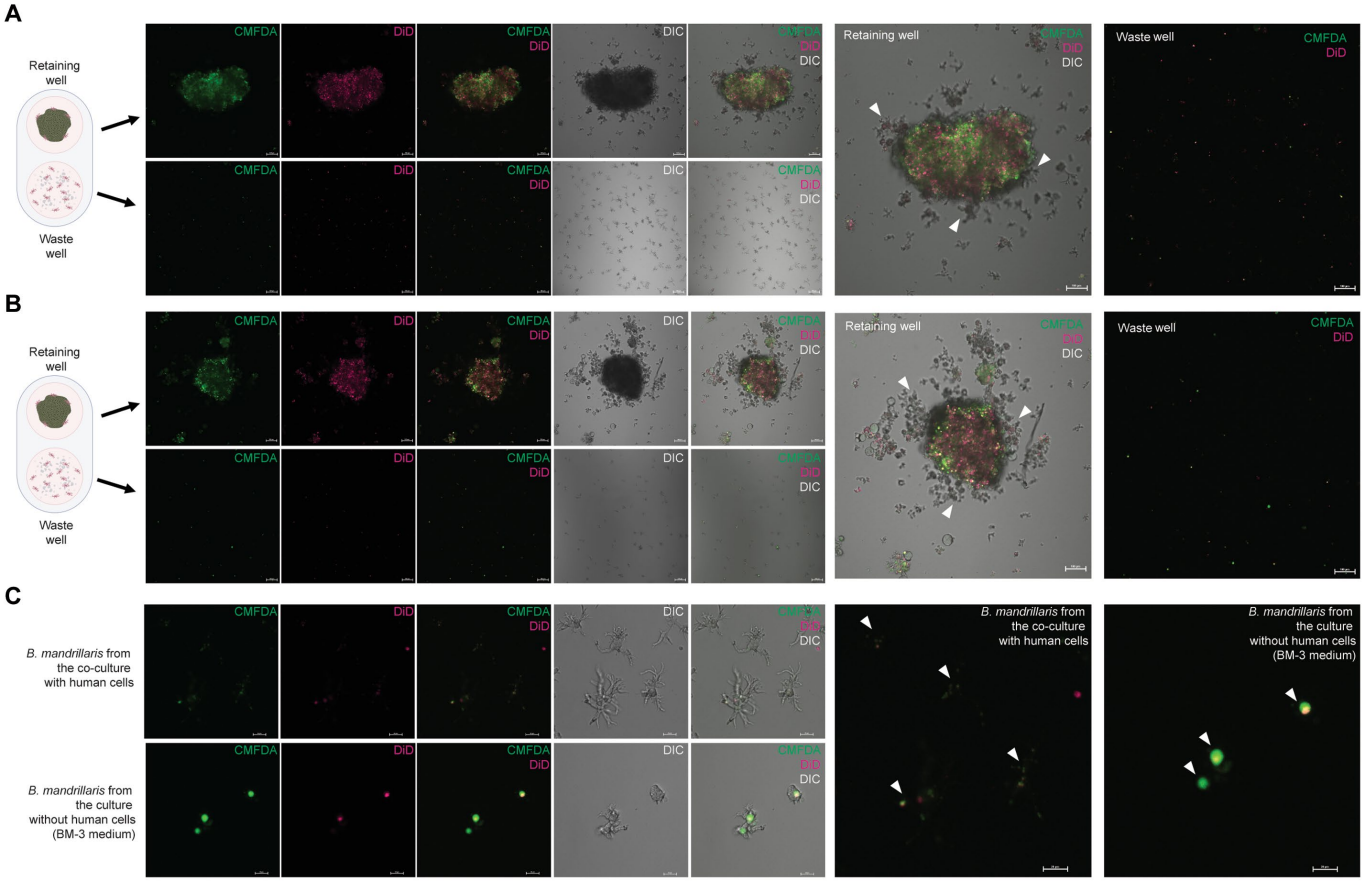
Cytophagy of the *B. mandrillaris* trophozoites in the 3DHSP. **(A)** Representative images of human neurospheroids cocultured with *B. mandrillaris* trophozoites obtained from culture with human neuroblastoma SH-SY5Y cells. Scale bars, 100 μm. **(B)** Representative images of human neurospheroids cocultured with *B. mandrillaris* trophozoites obtained from the feeder-free culture (BM-3 medium). Scale bars, 100 μm. **(C)** Zoomed-in images of *B. mandrillaris* trophozoites. The upper panels are representative images of the trophozoites obtained from **(A)**, while the lower panels are images of the trophozoites obtained from **(B)**. Microscopic images were captured in differential interference contrast (DIC) and fluorescence modes. The 3DHSP was tilted for 90 min. Scale bars, 20 μm. CMFDA, the protein-binding fluorophore, and DiD, the lipid-binding fluorophore.

To demonstrate the use of 3DHSP for elucidating the mechanisms of trophozoite survival, we observed host cell ingestion by trophozoites. Proteins and lipids of human neuroblastoma cells were labeled with the fluorophores CMFDA and DiD, respectively. A 90-min tilting was performed to obtain more trophozoites in the waste cells. Regardless of the sources, the trophozoites scattered throughout the retaining well (arrowheads in Figures 5A,B). Most of the trophozoites in the waste well were positive for CMFDA and DiD (Figure 5C), indicating human cell ingestion. After plate tilting, the trophozoites in the waste wells were observed under a laser confocal microscope. The SH-SY5Y-derived trophozoites had more elongated cytoplasm, while the feeder-free trophozoites were rounder with short protrusions of cytoplasm (DIC images, Figure 5C). Human lipid and protein were observed in the cytoplasm of the trophozoites in a distinct pattern. The SH-SY5Y-derived trophozoites had dispersed granules containing human protein and lipids (arrowheads in upper panel in Figure 5C). In contrast, larger vacuoles were observed in the trophozoites obtained from the feeder-free culture. These vacuole-like structures contained both human lipids and proteins (arrowheads in upper panel in Figure 5C). These results imply that the amoebae obtained the energy source from a human neurospheroid in a different way.

## 4. Discussion

Forming the neurospheroid in a hanging drop of the 3DHSP allows assessment of the cytotoxicity of *B. mandrillaris* trophozoites, a clinical isolate of the parasitic amoeba. The 3DHSP offers advantages for host–parasite interaction in a context relevant to the 3D organizing cells in a given tissue. In the hanging drop, the *B. mandrillaris* trophozoites decreased the viability of human cells in the neurospheroid. The 3DHSP is capable of separating the amoebic trophozoites from the neurospheroid, allowing accurate measurement of host and parasite survivability. Following separation, it is also feasible to study the mechanism of host cell ingestion of the amoeba. Although the separation efficiency of trophozoites and amoebas needs further improvement, the dual phenotype platform is likely applicable for screening a lead compound that has low cytotoxic and amoebicidal effects in a single plate.

Several strains of *B. mandrillaris* have been isolated from patients and from the environment across continents. *B. mandrillaris* trophozoites can be maintained in a standard laboratory as a routine procedure, allowing the study of their pathogenicity (Greninger et al., 2015). Here, the *B. mandrillaris* strain was isolated from the third case of *Balamuthia* amoebic encephalitis in Thailand (Intalapaporn et al., 2004; Krasaelap et al., 2013) and used as a representative virulent strain (Law et al., 2023). We have been able to grow this amoeba strain in feeder-free conditions using BM-3 medium in addition to coculture with human lung carcinoma A549 and neuroblastoma SH-SY5Y cells (Pengsart et al., 2022). Regardless of the culture conditions, the *B. mandrillaris* trophozoites exert a cytotoxic effect on the human neurospheroid.

A decrease of neurospheroid size was observed in the presence of *B. mandrillaris* originating from BM-3 medium in both tilted and non-tilted plates. However, the size of neurospheroid cocultured with *B. mandrillaris* originating from human feeder cells decreased only in the tilted plates. This difference is likely due to rapid damage of neurospheroid caused by the trophozoite derived from the BM-3 medium. Moreover, there was discrepancy in the data of DNA detection and ATP measurement in this study. In Figure 4B, a factor of difference of 600 was observed at 45 min in the waste well in the tilted plated in comparison to the non-tilted plate. This difference is much higher than in the other conditions, especially, at 90 min in the same condition, where only a factor of difference of 20 was observed. This difference between 45 min and 90 min for spheroids incubated with *B. mandrillaris* originating from human cells was not in agreement with the ATP results in Figure 4A. This discrepancy issue might be due to sensitivity and nature of the parameter detected. The amount of DNA represents dead and alive cells (Cangelosi and Meschke, 2014) while ATP levels are dependent on metabolic activity of cells (Chan et al., 2013).

However, there is a noticeable question regarding which culture to use. The trophozoites harvested from the coculture with human SH-SY5Y neuroblastoma cells cannot be used until the monolayer of human cells is removed from the culture dish. Moreover, the trophozoites obtained from coculture with human SH-SY5Y cells tend to form cell clumps in the hanging drop of the 3DHSP, limiting the separation of trophozoites from the neurospheroid. Thus, optimizing the tilting time of the 3DHSP is recommended. In contrast, our results showed that the BM-3-derived trophozoites could be separated from the neurospheroid at a higher efficiency in the 3DHSP. No cell clump was observed when culturing the BM-3-derived trophozoites in the hanging drop on the 3DHSP. Due to the ease of cell preparation, the feeder-free culture in BM-3 is suitable for coculture with neurospheroids. Nevertheless, BM-3 medium contains many nutrients and is not commercially available (Schuster and Visvesvara, 1996). Thus, the use of BM-3 medium is limited. The reason underlying the clump of the trophozoites remains unknown. It is likely that trophozoites tend to form clumps when lacking nutrient sources or in response to changes in the microenvironment. However, this opens a question that needs to be further elucidated since this cell clump might also be a mechanism of drug resistance, as observed in quorum sensing of bacteria (Donabedian, 2003; Bassler and Losick, 2006).

Physiologically relevant models are essential for validating the therapeutic effect of lead pharmaceutical substances. It is also feasible to use immunodeficient and immunocompetent mice to study the pathogenesis of granulomatous encephalitis (Janitschke et al., 1996) and the route of infection (Kiderlen and Laube, 2004). However, the use of animal models in drug screening is time-consuming and expensive and is possible only in laboratories with standard animal care. The culture of brain tissue or neurons may recapitulate *in vivo* conditions. A drug screening study used human brain tissue explants to indicate the therapeutic effect of nitroxoline (Laurie et al., 2018), leading to successful treatment of *Balamuthia* amoebic encephalitis in a human patient (Spottiswoode et al., 2023). Thus, a greater degree of physiological relevance allows higher similarity between *in vitro* and *in vivo* assays. Nevertheless, the *in vitro* culture of human brain tissue faces several concerns, such as tissue shortage, limited number of cells, and difficulty in culture and expansion. Cancer cell lines are more convenient for culture and expansion, supporting high-throughput drug screening. Human neuroblastoma SH-SY5Y cells originate from immature neurons (Biedler et al., 1978; Gilany et al., 2008). However, *in vitro* models of human neuroblastoma SH-SY5Y cells are applicable for elucidating the mechanisms of neuron dysfunction (Elyasi et al., 2020). Therefore, the use of neuroblastoma cells reduces the need for primary neurons and is more suitable for drug screening. After drug screening in the cancer cell-based platform, the use of brain tissue explants or cerebral organoids is more relevant to the brain and can be used as a secondary screening phase without animal models.

Several cell culture platforms allow the generation of spheroids, including the embedding of cells in semisolid medium. A limit of the semisolid platform is the physical barrier against trophozoite penetration. Thus, a liquid drop allows cell contact without the physical barrier. Similar to other spheroid models, 3DHSP-derived neurospheroids have a hypoxic core (Anada et al., 2012; Leek et al., 2016). The advantage of hanging drop-based spheroid formation is the lack of dependence on a scaffold. Nevertheless, it remains difficult to capture images of a spheroid in a drop using an inverted microscope. Moreover, the drop is prone to evaporation, requiring a humid environment. Due to the tight adherence of trophozoites to the neurospheroid, it was difficult to separate the trophozoites from the neurospheroid. Moreover, there were trophozoites surrounding the neurospheroid without cell contact after the 3DHSP was tilted. The sizes of trophozoites in the retaining well and the waste well were similar, implying that the trophozoites could be drained out from the retaining well. The presence of trophozoites in the retaining well might suggest a need to increase the flow but not the pore size.

Originally designed to assess the cytotoxic effect of CAR T cells against cancer spheroids (Chen et al., 2022), the 3DHSP is unsuitable for separating trophozoites from neurospheroids due to cell-to-cell interaction (Pengsart et al., 2022; Pengsart and Kulkeaw, 2022); however, it may be useful in a drug screen for assessing both cytotoxic and amoebic effects by adjusting the size of the neurospheroids and using the ATP of trophozoites as a readout, despite the low separation efficiency, thereby reducing interexperimental variation (Iversen et al., 2006; Larsson et al., 2020). However, improvements are needed for the use of 3DHSPs with fluorescence-or chemiluminescence-based plate readers for ATP measurement and high-content imaging for measuring the size of the neurospheroids.

## Data availability statement

The original contributions presented in the study are included in the article/Supplementary material, further inquiries can be directed to the corresponding author.

## Ethics statement

*Balamuthia mandrillaris* trophozoites were isolated from biopsied brain tissue of a human subject who gave written informed consent to participate (COA no. Si806/2020). The experiments involving *B. mandrillaris* trophozoites were approved by the Siriraj Institutional Review Board (COA. no. 146/2022). Ethical approval was not required for the studies on human cells obtained from commercially available cell lines in accordance with the local legislation and institutional requirements because only commercially available established cell lines were used.

## Author contributions

NW, WP, and KK: conceptualization. NW, WP, ZC, and SH: methodology. NW and WP: writing—original draft preparation and visualization. KK, ZC, and SP: writing—review and editing. KK and SP: supervision. KK: funding acquisition. All authors have read and approved the published version of the manuscript.

## Funding

The research project was supported by the Siriraj Research Fund, Grant no. (IO)R016433023, Faculty of Medicine Siriraj Hospital, Mahidol University and Industrial Strategic Technology Development for the program-development of disease models based on a 3D microenvironmental platform mimicking multiple organs and evaluation of drug efficacy (grant no. 20008413) funded by the Ministry of Trade, Industry and Energy (MOTIE, Korea). KK is supported by a grant for Mid-career Talented Researchers provided by the National Research Council of Thailand and Mahidol University (Grant no. R016541029).

## Conflict of interest

The authors declare that the research was conducted in the absence of any commercial or financial relationships that could be construed as a potential conflict of interest.

## Publisher’s note

All claims expressed in this article are solely those of the authors and do not necessarily represent those of their affiliated organizations, or those of the publisher, the editors and the reviewers. Any product that may be evaluated in this article, or claim that may be made by its manufacturer, is not guaranteed or endorsed by the publisher.
