# Supplementary figures and images for: Phenotypic assay for cytotoxicity assessment of Balamuthia mandrillaris against human neurospheroids
Source: Front Microbiol. 2023 Sep 5;14:1190530. doi: 10.3389/fmicb.2023.1190530 (PMC10513763; doi:10.3389/fmicb.2023.1190530)

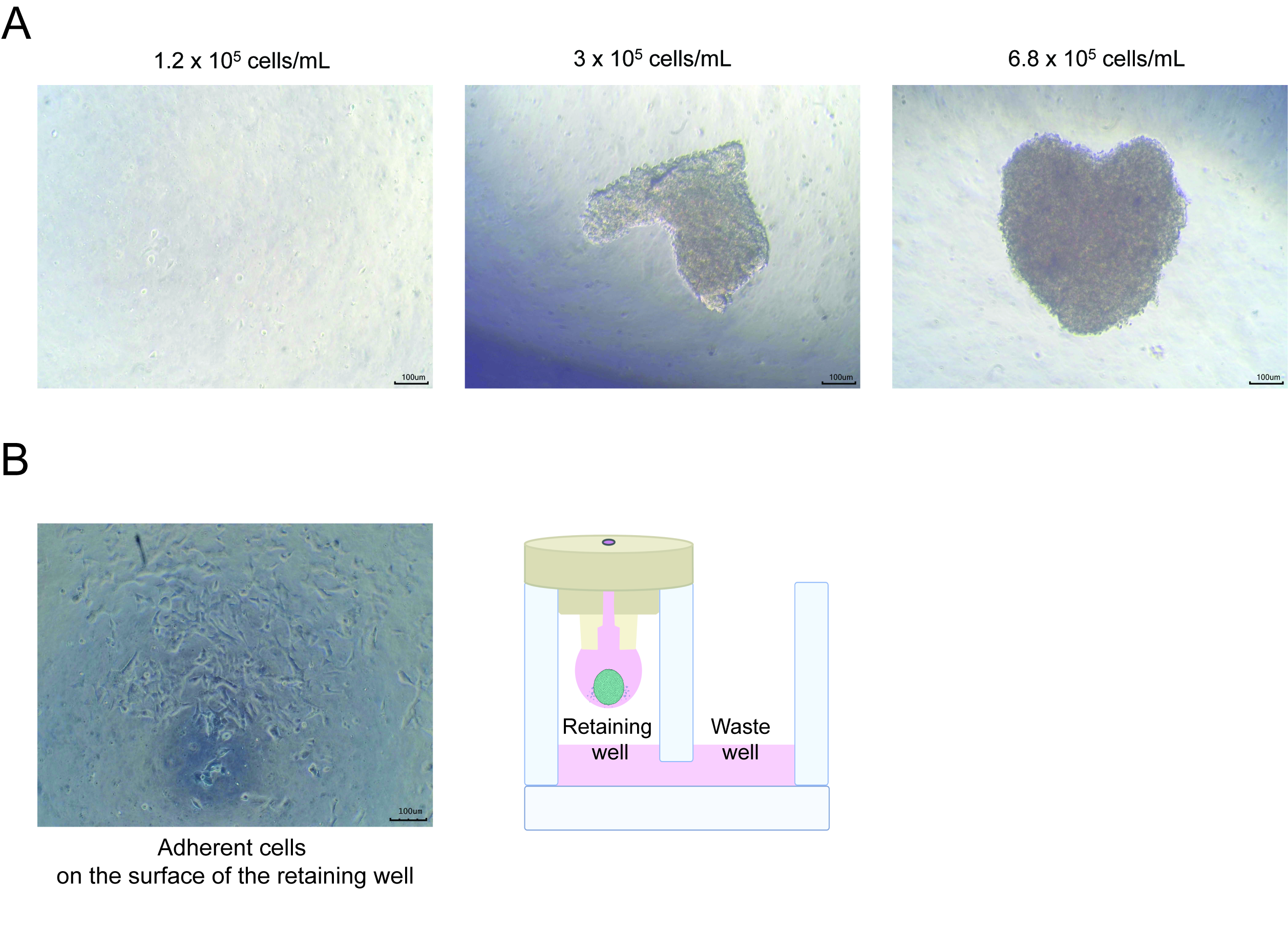

Supplement: Supplementary file 1 [file Image_1.TIF]

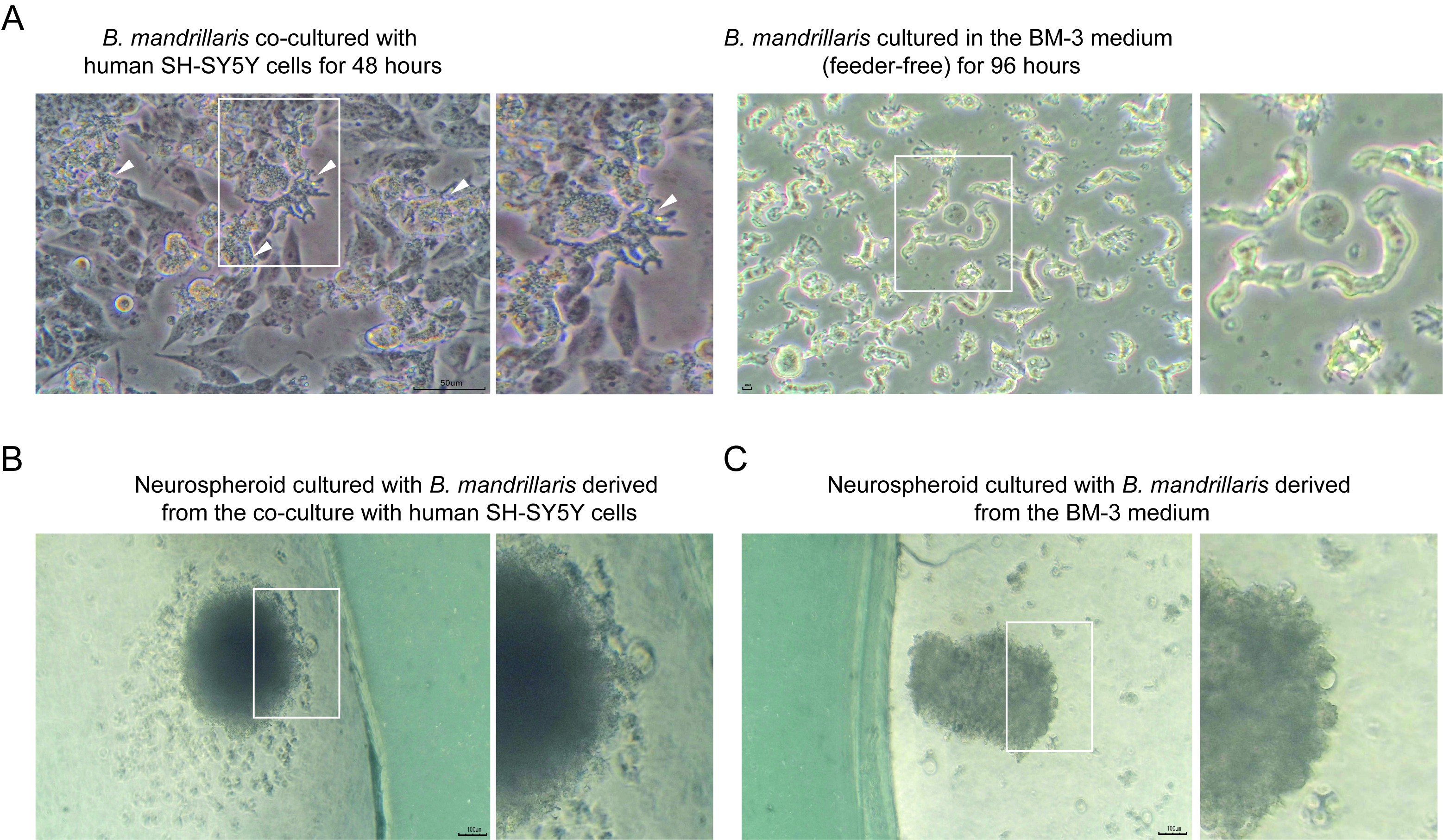

Supplement: Supplementary file 2 [file Image_2.TIF]
